# Harnessing information technology to improve women’s health information: evidence from Pakistan

**DOI:** 10.1186/1472-6874-14-105

**Published:** 2014-09-04

**Authors:** Rubeena Zakar, Muhammad Z Zakar, Shazia Qureshi, Florian Fischer

**Affiliations:** 1 Institute of Social and Cultural Studies, University of the Punjab, Lahore, Pakistan; 2 Faculty of Law, University of the Punjab, Lahore, Pakistan; 3 Department of Public Health Medicine, School of Public Health, Bielefeld University, P.O. Box 100 131, 33501 Bielefeld, Germany

**Keywords:** Health information, Information communication, Women’s health, Pakistan, Rural women

## Abstract

**Background:**

More than half of Pakistani women are illiterate, marginalized, and experience myriad health problems. These women are also disadvantaged in terms of their restricted mobility and limited access to public space. Nonetheless, user-friendly information and communication technologies (ICTs) have opened up new opportunities to provide them with information that is essential for their health and well-being.

**Methods:**

We established an Information and Communication Centre (ICC) in a village in Sialkot (Pakistan) on a pilot basis in 2009. The basic philosophy of the ICC was to provide women with health-related information by exposing them to modern sources of information on their doorstep. By design, the ICC was a community-based and community-managed institution where women could access information through online (e.g., internet, mobile phone etc.) and offline (e.g., CDs, TV etc.) resources. The ICC was managed by a group of local volunteer women who had the capacity and skills to use the devices and tools of modern ICTs.

**Results:**

We noted an overwhelming participation and interest from local women in the activities of the ICC. The women wanted to receive information on a wide range of issues, from family planning, antenatal care, and childcare to garbage disposal and prevention of domestic violence. Overall, the ICC was successful in initiating a meaningful “information dialogue” at community level, where much-needed information was retrieved, negotiated, mediated, and disseminated through intimate and trusted relations.

**Conclusion:**

We conclude that ICTs have the capacity to cross the barriers of illiteracy and can reach out to disadvantaged women living under a conservative patriarchal regime.

## Background

A majority of Pakistani women is illiterate and dependent on their male guardians (e.g. father, husband, brother, son) for seeking health care or accessing the resources and information needed to maintain their health [1]. With the exception of a tiny privileged minority, women’s social role is largely restricted to household chores. Therefore, they have limited exposure to or contact with the outside world [2,3]. Furthermore, these women cannot take independent and autonomous decisions on issues related to their reproductive and general health [4]. Additionally, due to the under-developed health care system and insufficient transport and communication facilities, rural women cannot get appropriate antenatal, delivery, or postnatal care services. As a result, there are high rates of maternal mortality and morbidity, especially among women of low socio-economic status [5]. The results of the Global Burden of Disease study 2010 indicate the relevance of maternal and neonatal disorders in terms of disability-adjusted life years (DALYs) in Pakistan. The DALY is a summary measure of population health, which describes the overall disease burden, expressed as the number of years of life lost due to premature mortality (YLL) and the years of life lived with disability (YLD). In Pakistan, maternal and neonatal disorders accounted for 11.9% of the total age-standardized DALYs. In comparison, DALYs attributable to maternal and neonatal disorders in countries of the developed world accounted for only 2.5% of the total age-standardized DALYs in 2010 [6]. Being relatively socially powerless, most of these women are vulnerable to various types of abuse and human rights violations, including sexual exploitation, spousal violence, and denial of reproductive freedom [7].

Given the serious deficit in their literacy and numeracy skills, the possession and independent use of information and communication technology (ICT) equipment (especially mobile phones, computers) are still considered to be a “man’s job” and women are incapable or reluctant to operate such equipment [8]. A combination of these factors can severely restrict women’s ability to access and use innovative information for the improvement of their health and well-being.

### Women, ICTs and empowerment

Recent advancements and popularization of ICTs, especially mobile phones, text messages and emails, have opened up new opportunities to upgrade the level and quality of information for marginalized women [9]. These technologies are inexpensive, user-friendly, and are also available in remote regions [10]. Because of technological innovations, ICTs can reach out to poor and socially excluded rural women by crossing the barriers of infrastructural underdevelopment, illiteracy, and the many restrictions and inhibitions imposed by the rigid patriarchal regime [11].

Nonetheless, the mere provision of ICTs may not be helpful in creating information equality or empowering marginalized women. Research has reported that some developing countries have successfully harnessed ICTs to expedite the process of women’s capacity building [10,12-14] and their involvement in income-generating activities. Although technology opens up new opportunities, it has limitations: ICTs cannot automatically create information equality or cater to the information needs of socially disadvantaged women. Technology, not being gender neutral, cannot itself guarantee empowerment [15]; rather, it could further widen the digital divide [10,16,17].

### Women’s access to health information

Studies have reported that, under conservative patriarchal regimes, any initiative meant to empower women is received with suspicion and skepticism [18]. In rural areas of Pakistan, women are usually denied access to information [10] and the information they do get is scrutinized and controlled by their male guardians. If they get any information at all, it might not be relevant or applicable to their real life because of their multiple dependencies on men. Furthermore, women lack economic resources and social support to try innovative initiatives.

In developing countries, one of the most crucial areas of women’s lives is the availability of relevant and timely information about their health and disease prevention [19]. In Pakistan, both traditional and modern medical systems coexist [20] and women receive health-related information from both traditional and modern sources [21-24]. The sources of traditional medical information include family elders, traditional birth attendants (TBAs), spiritual healers, and *hakeems*[21]. The sources of “modern” medical information include officials of the government’s health department, doctors, paramedics, community-based lady health workers (LHWs), TV, radio, advertisements for pharmaceutical products, etc. Since traditional and modern sources of health-related information represent different world-views and bodies of knowledge, sometimes the information provided appears to be contradictory. This adds to confusion among the end-users (women) [21-23]. There seems no effective and viable institutional mechanism to clear the confusion and cater to the women’s health-related information needs [22].

### Rational and aims of research

There are challenges and complications in providing health-related information to Pakistani women. Despite the overflow of information, especially through cable TV channels, Pakistani women are particularly disadvantaged in terms of accessing information that is relevant and applicable to their health-related issues. The problem is further complicated because of women-specific cultural restrictions. For example, women are discouraged from talking openly about their reproductive health issues as these are considered to be “women’s problems”.

Additionally, some women’s health conditions are stigmatized, such as sexually transmitted infections and abortion. Therefore, they are reluctant to talk about these topics. In such a conservative environment, it is not easy to upgrade the level and quality of health-related information for rural women [25]. Nonetheless, recent advancements in modern information technology have opened up new avenues to reach out to these women by cutting across cultural, structural, patriarchal, and geographical boundaries [10,12-14]. Particularly, cell phones have increased women’s accessibility and connectivity because of affordability and usability. Providing information using ICTs is mainly used to mobilize the community to solve their own health problems [22]. However, there is paucity of literature that describes how this approach could be beneficial for rural women within their cultural, structural and economic contexts.

This paper intends to document the experiences gained from the establishment of an Information and Communication Center (ICC) in improving the level of health-related information of rural women in Sialkot, Pakistan. This paper also evaluates the capacity of ICC in improving the level of health-related information by conducting a cross-sectional survey with women who directly or indirectly sought information from the ICC.

## Methods

This study was conducted in two phases: during the first phase, an ICC was launched at community level, and during the second phase, we evaluated the functioning of the ICC by conducting a cross-sectional comparative study among three groups of women. The study methods were reviewed and approved by the Institutional Review Board (IRB) of the University of the Punjab.

### Phase I: Establishment of the Information and Communication Center (ICC)

#### Objectives of ICC

Despite the various advantages of ICTs, these alone cannot bring about social change [15]. Arguably, technology needs social institutions that help individuals to set the direction and orientation of change in a particular direction. Keeping this assumption in mind, we decided to establish an ICC at the community level.

The ICC was designed to help women to use ICTs to improve and update their information within their socio-cultural, structural, and capacity constraints. The primary objective of the ICC was to mobilize the community’s resources, especially the social capital, to augment the capacity of rural women to seek the information they desired within their own locality. The specific objectives of this ICC were:

•To build the capacity of women by developing an information-seeking culture and exposing them to diverse sources of information including ICTs;

•To enhance coordination and interaction between local health care functionaries and women by connecting them to the latest information at the ICC; and

•To retrieve the latest information about women’s health using modern ICTs and disseminate that information to women using simple local language and concepts, and to create an information-seeking and information-utilization culture.

#### Process of ICC

During the whole process, the research team undertook the role of interventionists, who not merely helped the women (facilitative role), but taught the women how to help themselves (expert role) [26]. The planning process of ICC entailed seven stages which are defined less as temporal sequence than as logical areas where the interventionists worked [26]. These stages were: 1) Contacting local people, both ordinary and influential to get familiarity with the community. 2) This familiarity helped to define relations with the community. At this stage, we also entered into a sort of contract; it was both a formal as well as cognitive contract with the community’s women. 3) Delineation of the study setting. 4) Data collection to make evidence-based diagnosis of the specific problem (the gap between actual availability of information and the information needs of the women). 5) Based on the diagnosis, the intervention (ICC) was designed and implemented. 6) When local women started getting integrated with the intervention and realized its utility and relevance, our involvement gradually reduced. 7) At the last stage, the intervention stood terminated from our side; as the local women owned the intervention and started operating it by themselves as a part of the local information system.

#### Structure of the ICC

By learning from our previous experience of establishing an ICC to assist the capacity building of farmers in 2006 [11] we decided to establish another ICC to improve women’s access to health-related information. The ICC was established in a village named *Pathanwali* in Sialkot (Pakistan) from January to December 2009 on a pilot basis. After the launch of a campaign for community mobilization, one influential villager in *Pathanwali* volunteered his premises to establish the ICC. It was a big room that could accommodate about 60 women at a time and had some open space in front of it. The premises already had an electricity supply and one phone line. Three computers with internet connections and one multimedia projector were installed in the room. The ICC was managed by a group of volunteer women who had the capacity and skills to use the tools and devices of modern ICTs such as computers, the internet and mobile phones.

In addition to these volunteers, we also invited local health care provision staff to participate in the ongoing activities of the ICC. The participation of local health personnel had multiple benefits: 1) they could update their own information using ICTs; 2) the local women had some familiarity with the staff, so the information they shared at the ICC carried some validity and authenticity; and 3) by creating awareness and interest, they could also facilitate the local women to participate in ICC activities.

The ICC remained open for about three hours per day. Timings were not rigidly fixed and depended on the availability of electricity, the weather, and the household engagements of women. Normally, it was opened from 10:00 a.m. to 12:30 p.m. (when children were in school) and 3:00 p.m. to 6:00 p.m. There was no restriction or qualification for membership of the ICC. All women living in the village and nearby villages could be members; no membership fee was levied. Due to cultural reasons, men were not allowed. However, male health care providers could be members and could attend ICC proceedings by invitation. Women from ethnic and religious minorities and marginalized sections of the community were particularly encouraged.

#### Philosophy and functional parameters of the ICC

The basic philosophy of the ICC was to improve rural women’s information regarding health-related issues by exposing them to modern sources of information on their doorstep. The ICC was a community-driven and people-owned institution. It was of the community, for the community, and by the community. By design, this institution was meant to retrieve the latest information from online (e.g., internet, mobile phone) and offline (e.g., CDs, TV) ICT sources and transmit it to the local women through face-to-face interaction and dialogue. The idea was that, under the umbrella of the ICC, information would be shared, explained, and interpreted to ordinary women through culturally understandable social discourse. It may be noted that the intended role of the ICC was not just to transmit information, but also to initiate a social process involving the information retrieved through ICTs by harnessing women’s local contacts and web of relations. In this way, women could have the opportunity to evaluate the applicability, relevance, and economic viability of a piece of information within their local resources and in the cultural context. In essence, the ICC was meant to create information plurality at a community level by connecting relatively more informed and less informed women and encouraging them to use ICTs to upgrade their information and awareness about their own and their families’ health-related issues.

At the ICC, information was retrieved, shared, and discussed with rural women by various stakeholders at community level. The stakeholders included health professionals such as doctors, nurses, LHWs, TBAs, and other service providers at the local level. It may be noted that information-based dialogue was not restricted to health professionals but also included local agriculture extension officials, school teachers, and community representatives. All of these officials/social actors negotiate and influence the local population to accept or reject a particular piece of information. Therefore, all of them were encouraged to be associated with the ICC to initiate a comprehensive dialogue with ordinary women. For example, they discussed the causes and prevention of different infectious diseases such as malaria, dengue fever, hepatitis, etc., antenatal and postnatal care, pregnancy complications, and childbirth with women and their families.

**Figure 1:**
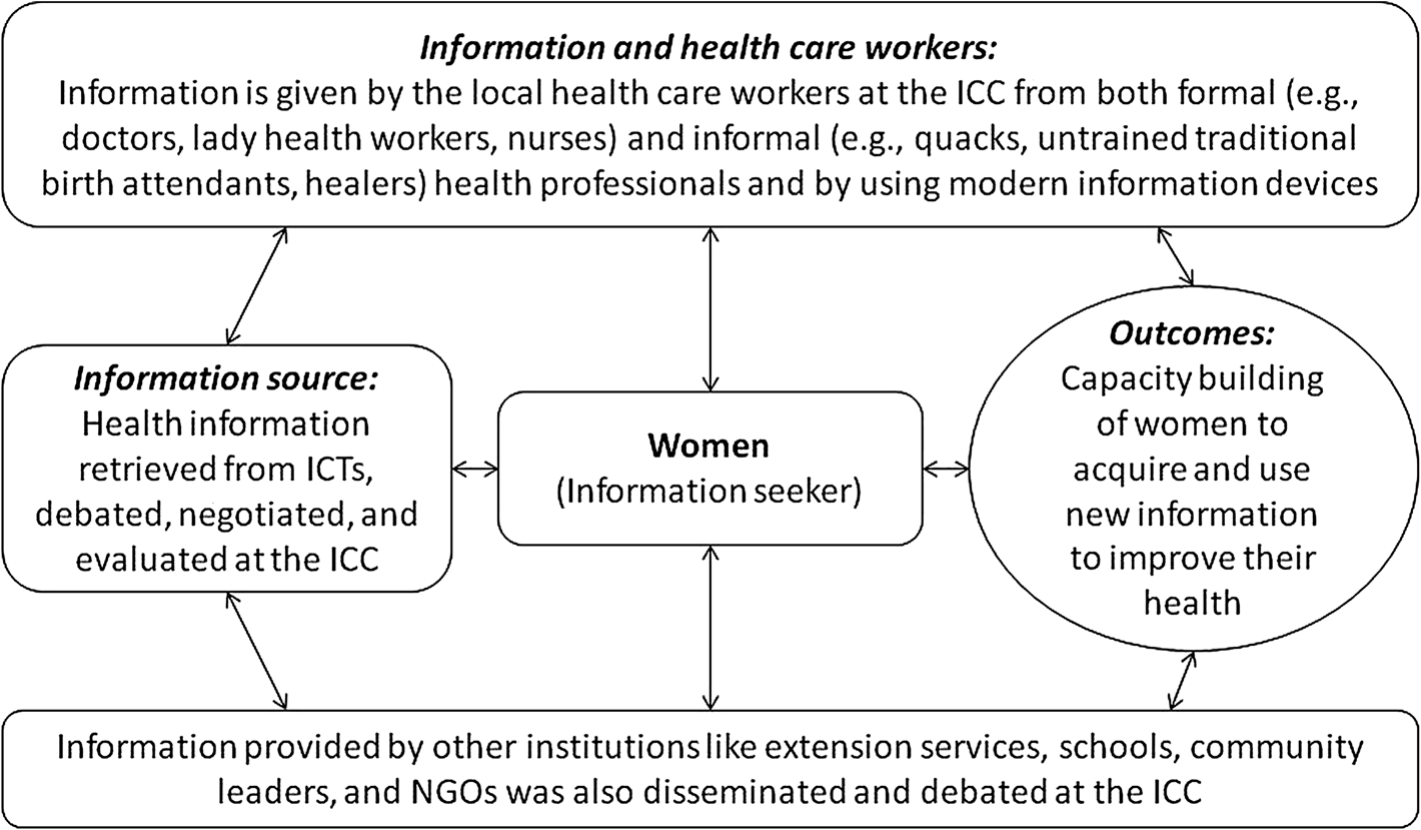
Functioning of the Information and Communication Center (ICC) at community.

The ICC was in fact designed to mobilize and coordinate the existing community health information resources to build the capacity of women to seek appropriate health care. By pooling various information resources, the ICC was designed to develop information-seeking cultures among women, especially the marginalized and illiterate ones, as they were not able to connect and engage with the new information systems. At the ICC a major focus was to keep the excluded women on board, include them in the ongoing information discourse and create information equality. In addition to giving them exposure to the benefits of ICTs, we also wanted to develop their connectivity and face-to-face interaction with community-level health care functionaries like LHWs, family planning staff and *dais* (traditional birth attendants), who were also members of the ICC (see Figure 1). The purpose of bringing information haves and have-nots together under the roof of the ICC was to initiate discourse around health-related information.

#### Data collection

Qualitative data was collected regularly during the functioning of ICC with participants/attendees of the ICC. The data were collected by using techniques of field observation, informal interviews, and in-depth interviews with women. Notes were taken from the ongoing conversation of women with the research teams and other stakeholders at the ICC. These notes captured the perspective of women regarding their experiences with the ICC, their information needs and the relevance and practicality of information, as well as their expectations from the ICC activities. At the end of each month, the data was compiled and discussed with all team members during the debriefing sessions.

After the completion of eight months of the ICC functioning, the data was translated into English for analytical purposes. Constant comparison strategies were used by systematic examination of similarities between the different groups of data. After multiple readings of the transcripts common themes were identified. The themes were coded into categories [27]. The data were searched for similar or repeating ideas and finally four themes emerged from the data: 1) women’s connectivity with information agencies; 2) creating sense of empowerment among women; 3) cross-verification of information; and 4) getting non-health information.

### Phase II: Evaluation of the functioning of the Information and Communication Center (ICC)

The ICC operated for about one year. Initially, the community responded with caution and reservations. Some people were suspicious about the “hidden purposes” of the ICC. Some started floating conspiracy theories such as “ICC advances a Western agenda to promote birth control”. Others considered the ICC to be some foreign-funded organization aiming to “secularize Pakistani society” under the guise of women’s health. Nonetheless, gradually, with the increased participation of the local women, these suspicions and fears subsided.

#### ICC evaluation and capability approach

The intervention launched at the ICC was evaluated within the framework of capability approach [28] introduced by Sen [29] and others [30,31]. A number of studies have referred to the relationship between ICT and capability approach [32,33]. In this framework [29], expansion of freedom was viewed “both as the primary end and as the principal means of development” (xii). Arguably, both political and social freedom is contingent upon individual’s freedom to access information of his/her own choice. Hence, for many advocates of capability approach, access to information is a basic human right. For the optimal functioning, one needs to stay healthy; and to be healthy access to health information is necessary.

#### Evaluation process of functioning of ICC

We noted that women from almost all sections of the village participated in the ICC’s activities. However, to measure the success of the ICC caused some difficulties, because information seeking and utilization entails a complex social process. The functioning of the ICC was evaluated by conducting a descriptive comparative study in selected villages of Sialkot. For evaluation purposes, we selected women of reproductive age (15–49 years) who had ever been married living in the catchment areas of the ICC. Three group of women were selected: firstly, women who had direct contact with the ICC (i.e., women who attended the information sessions at the ICC and participated in most of its activities), secondly, women who had indirect contact with the ICC (i.e., women who did not actually participate in ICC activities but received information indirectly from women who visited the ICC), and thirdly, women who had no direct or indirect contact with the ICC.

Out of the total number of ICC participants (n = 2,045), a random sample of 380 women was selected. The same number of women was randomly selected from those who had indirect contact with ICC participants and those who had no contact with the ICC. During the ICC activities, the women who attended the information sessions at ICC were regularly asked to identify the women in their personal networks with whom they had contacted and shared the information received from the ICC. The researcher at the ICC prepared a list of all those women who did not actually participate in the ICC activities but received information indirectly from women who visited the ICC, that is, the indirect group. And women were selected randomly from the list to conduct interviews. For the selection of no-contact groups of women, LHWs of the localities situated in the catchment areas of ICC were contacted. LHWs are community based female health workers responsible for providing preventive care to the local women at their doorsteps. They live in the same communities and have the knowledge about all women of their localities. The LHWs went door to door to get information and prepared the list of all women who did not participate in the ICC activities. The no-contact group of women was randomly selected from this list. The response rate was 90%, 87%, and 74% among direct, indirect and no contact group, respectively.

#### Data collection

The data were collected by using a face-to-face cross-sectional survey method at the end of a one year time period after the ICC’s establishment, between January and April 2010. To assess the impact of the ICC project on the health information behavior of women, a structured pre-coded questionnaire was used to collect information. The interview schedule was developed on the basis of information provided and discussed at the ICC (e.g., information about reproductive health, pregnancy, childcare, garbage disposal, prevention of domestic violence). The interview schedule was developed on the basis of information provided at the ICC. Each variable had some indicators to measure the information about these variables. For example, the information regarding availability of contraceptives was measured by asking if women knew the free of cost availability of contraceptives at basic health units, family planning clinics, and family welfare centers. Data were collected by three female field researchers. They were involved in ICC activities and had a prior experience of survey research. Additional intensive data collection training was provided to field researchers before data collection. Written informed consent for participation was obtained from all study participants.

#### Data analysis

Data were analyzed by using SPSS version 17. A bivariate analysis was performed by comparing the information gathered from the three groups of women using a Chi-square test of independence.

## Results

### Functioning of the ICC in the local context

At the ICC, we noted that women took an interest in seeking information from diverse sources depending on their social capacity and specific information needs. For instance, illiterate women sought information about reproductive health from local *dais* (birth attendants) or LHWs or from the neighboring women. At the ICC they tried to verify the validity of that information. Other local institutions like schools, mosques, the basic health unit (BHU), vaccinating staff, and even veterinary staff were also providing information about various health-related issues. We noted that, for women, getting information was not a problem; the real problem was how to get relevant, applicable, and practically viable information.

#### Connecting women with various information providing agencies

In medical encounters, there has been a chronic complaint of a disconnection between information providers' and information receivers’ perspectives. Ideally, the doctor-patient relationship is structured in such a way that the patient can get appropriate medical information from the doctor. Nonetheless, in the rural areas of both developing [19,24] and developed countries [20,34], patients feel that their concerns are not heard or responded to in a manner that meets their needs.

It was noted that most of the women came to the ICC to verify information and make sense of it within their cultural and cognitive context. From the queries and comments of the women, we found that there was a sort of disconnect between the information provided by various government functionaries and the possibilities of women actually utilizing this information. For instance, many women considered that information from government institutions about “flood warnings” or use of “contaminated water” or “family planning methods” was useless, irrelevant, and not applicable to their life-world. For example, one woman in her early forties said:

‘They [referring to staff of the department of population welfare] are repeating that we should use contraceptive pills. But we heard and witnessed that these pills are dangerous (by using these pills, the user becomes over-weight). Nobody explains things to us; when we put questions health care staff just laughs at us.’

Similarly, many women knew that treatment from medical quacks or self-medication was risky but they still tried these options. The reason was that the quacks translated and interpreted modern medical information according to the cognitive capabilities of village women. At a local level, it was not easy to compete with quacks on the “information turf” and women usually subscribed to the quacks’ version of medical knowledge while ignoring the scientifically valid information provided by the government health department.

The most effective and engaging meetings at the ICC were those when local health care staff, community workers, and common women got together, talked, and discussed various health issues. The health care staff discussed local knowledge about symptoms and the prevention of common childhood diseases and the risks of self-medication by using pictures and videos on computers and the internet. The LHWs and TBAs helped to translate the technical information provided by health experts into local women’s concepts and world-view about health and illness. This face-to-face interaction at the ICC played an important role in debating the relevance and applicability of information in the local cultural context. Participant women were also concerned about the absenteeism of health care staff and the irresponsible and dangerous behavior of medical quacks (in villages about 70% of women sought health care from various types of medical quacks). The most frequent complaints were about the uncaring and unsympathetic attitude of the health care staff, especially towards poor women.

#### Creating sense of empowerment among the women

One of the most important and unexpected impacts of the functioning of the ICC was a sense of empowerment among ordinary women. Since officials from various government departments interacted with women and answered their questions at the ICC, women felt satisfied and empowered as otherwise the health care staff rarely took ordinary women’s concerns seriously. It appeared as though the “collective” power of the ICC made medical staff accountable and answerable to ordinary women. One 30-year-old woman while explaining her feelings said:

‘These uncaring people [referring to health care staff] never listen to us when they are in their offices as they know that we don’t have any say. But when we are here [referring to the ICC] they answer our queries very seriously.’

#### Cross-verification of medical information

During our interaction with women at the ICC we noted that they were not interested in the information given by the health department on the prevention of hepatitis C and other standard one-way flow of information. Women needed new information but they wanted to ensure that it was applicable to their local needs and available resources. They tried to verify information from their own trusted resources. If information was successfully used by their neighbors, they were more likely to use that information. Although women were not information resistant, they were dissatisfied with the prevailing “information environment”. One middle-aged woman with no schooling said:

‘Everybody keeps on telling us what is healthy and what is dangerous: as if we are stupid. Nobody tells us how to live a healthy life within our available resources.’

With regard to the utilization of health care services, almost all the women knew that medical quacks were not fully qualified doctors. But despite knowing this, they sought medical care from quacks as they had no other option. One 40-year-old woman with five years of schooling said:

‘It’s not a question of information; it’s a question of affordability and availability. When we go to him [referring to a local quack], he helps us. He gives something to relieve the pain.’

#### Seeking information on non-health-related matters

Despite their illiteracy and social exclusion, almost all the women knew the importance of new information for the solution of their health and other problems. Although the main purpose of the ICC was to give information about health and health-related issues, women were curious to get information about other issues of social and economic significance like information about the employment of their husband or son or knowledge about an effective ointment for healing the nipples of their buffalo (about 45% houses had buffalo, cows, or goats on their farms). Women also needed information about minor issues like how to get rid of head lice, treatment for painful teeth, to reduce weight, or some easy remedy for their backache or to improve their children’s blood iron level.

Some of the women also sought information about foreign currency rates and about some foreign countries. The reason for this interest in foreign currency conversion rates was that the husbands or sons of some women in the village and catchment areas were working in the Middle East and various European countries. Their husbands/sons sent remittances, so women were interested in the foreign currency conversion rates and they thought that the ICC could give them correct information. A 30-year-old woman with no schooling whose husband was working in Saudi Arabia regularly asked about the conversion rate of the Saudi Riyal to Pakistani rupees. She suspected that her father-in-law did not tell her the true rate and gave her less money than her husband intended. Another middle-aged woman whose son was working in the USA as a taxi driver said: ‘*What does your computer say about today’s conversion rate for the dollar?’*

We noted that women from marginalized families were particularly vulnerable to disease and unhealthy living conditions because of their poverty, poor sanitation, and social exclusion. One woman said:

‘The information you give us is useless. We process animal dung to make fuel for kitchen use. We do cleaning jobs around the toilet [no concept of gloves]. Our animals [goats, hens, etc.] live with us and make things dirty. There is no sewerage and waste water flows around. We know the virtues of cleanliness, but we cannot afford it.’

Another woman with low socio-economic status said:

‘Yes, information is good, but how to generate resources… You tell us to boil water for drinking, but where is the fuel? We can hardly arrange fuel for cooking two meals a day… How can we boil water and then cool it for drinking when we don’t have a fridge?’

### Evaluation of the functioning of the ICC

After one year of the ICC functioning on a pilot basis, we evaluated its impact on the level of information of women who came into contact with the ICC directly, indirectly, or not at all. The age distribution was different in each group. Mostly young women participated in ICC activities. As shown in Table 1, young women (<25 years) were highest (55%) in the direct-contact group, followed by 48.4% and 21.5% in the indirect and no contact groups, respectively. Among the women who had direct contact with the ICC, 43% had no school education and 53.7% had a familial monthly income below 10,000 Pakistani rupees (equivalent to 120 US $). The majority of the women (direct contact = 79.6%, indirect contact = 81.6%, and no contact = 84%) were housewives and less than 20% were employed as skilled or unskilled workers.

**Table 1:** Socio-demographic characteristics of the sample (N = 1,140)

**Variables**	**Direct contact with the ICC, % (n = 380)**	**Indirect contact with the ICC, % (n = 380)**	**No contact with the ICC, % (n = 380)**	**Chi square p-value**^**a**^
Age of respondents (in years)				<0.001
<25	55.3	48.4	21.5	
25-34	25.3	31.6	32.6	
≥35	19.4	20.0	45.9	
Education of respondents				<0.001
No schooling	43.2	46.5	32.1	
Up to secondary level	37.9	35.3	42.1	
> Secondary level	18.9	18.2	25.8	
Education of the husband				<0.001
No schooling	40.1	34.7	32.6	
Up to secondary level	42.6	41.1	34.8	
> Secondary level	17.3	24.2	32.6	
Caste (*Barader*i)				<0.001
Gujjar	32.6	28.4	14.7	
Jatt	29.5	34.8	24.2	
Rajput	24.2	20.5	28.9	
Kashmiri	10.3	11.1	20.6	
Others	3.4	5.2	11.6	
Duration of marriage (in years)				<0.001
≤10	54.2	46,5	20.6	
11-20	26.6	32.7	34.7	
>20	19.2	20.8	44.7	
Women’s employment status				<0.001
Housewife	79.6	81.6	84.2	
Unskilled worker	15.2	16.8	4.7	
Skilled worker	5.2	1.6	11.1	
Total family income (in Pak Rs.)^b^				<0.001
<10,000	53.7	41.1	39,5	
10,000-20,000	26.7	38.4	19.4	
>20,000	19.6	20.5	41.1	
Number of children				0.57
≤3	46.8	50.5	48.0	
>3	53.2	49.5	52.0	
House goods available at home ^c^				
Radio/cassette player (yes)	40.1	44.7	38.4	0.18
TV (yes)	52.1	46.5	57.9	<0.01
Bicycle (yes)	42.6	34.7	37.3	0.07
Motorcycle (yes)	20.5	17.3	23.1	0.13
Car (yes)	3.1	2.1	3.6	0.83
Mobile phone (yes)	44.2	47.9	52.1	0.09
Computer (yes)	8.4	10	11.0	0.47
Internet connection (yes)	2.6	3.1	3.6	0.71

^a^Fisher exact test.

^b^Pak Rs. = Pakistani rupees; 1 US $ = 97 Pak rupees.

^c^Owned by husband/son/brother.

Regarding the availability of modern information devices at home, 44% of women in the direct contact group, 47.9% in the indirect contact group, and 52% in the no contact group reported having mobile phones at home. Most of the women reported that usually mobile phones were owned and used by male members of the household. Similarly, there was no difference in the availability of computers (p = 0.47) and internet connections (p = 0.71) at home in all three groups.

There was a significant difference in terms of having information regarding reproductive health issues among the three groups of women (see Table 2). About 97% of women in the direct contact group reported having information about the availability of contraceptive methods, compared with 82% of women in the no contact group (p < 0.001). Similarly, 89% of women in the direct contact group reported having information about dangerous signs during pregnancy. There was no significant difference among the three groups with respect to information about use of appropriate diet during pregnancy (p = 0.21) (Table 2).

**Table 2:** Knowledge/information about reproductive health (n = 1,140)

**Variables**	**Direct contact with the ICC, % (n = 380)**	**Indirect contact with the ICC, % (n = 380)**	**No contact with the ICC, % (n = 380)**	**Chi square**^**a **^**p-value**
**Information about:**				<0.001
Availability of contraceptives				
Yes	97.4	91.6	82.1	
No	2.6	8.4	17.9	
Proper use of contraceptive methods				<0.001
Yes	90.0	65.3	57.8	
No	10.0	34.7	42.2	
To get rid of misconception about contraceptive methods				<0.001
Yes	81.1	58.4	31.6	
No	18.9	41.6	68.4	
About antenatal care				<0.001
Yes	92.1	87.6	82.9	
No	7.9	12.4	17.1	
Dangerous signs during pregnancy				<0.001
Yes	88.9	57.8	55.0	
No	11.1	42.2	45.0	
Use of appropriate diet during pregnancy				0.21
Yes	82.1	77.9	77.4	
No	17.9	22.1	22.6	
Safe delivery				<0.001
Yes	88.9	58.4	53.9	
No	11.1	41.6	46.1	
Post-partum care				<0.001
Yes	77.8	37.4	51.8	
No	22.2	62.6	48.2	
Breast-feeding				0.09
Yes	91.8	87.4	87.8	
No	8.2	12.6	12.2	
Menstrual hygiene				<0.001
Yes	78.2	57.6	37.4	
No	21.8	42.4	62.6	

^a^Fisher exact test.

We also assessed women’s level of information regarding knowledge about general health issues (see Table 3). Information about the dangers of self-medication was better among the direct (76.6%) and indirect contact groups (77.1%) than the no contact group (66%). Similarly, regarding the use of disposable syringes for injection, 83% women in the direct contact group reported having this information, whereas there was no difference in the indirect (51.8%) and no contact (52.9%) groups. In contrast, there was no difference regarding information about causes of hepatitis B and C among the three groups (p = 0.12). Regarding garbage disposal, 53% and 49% of women in the direct and indirect contact groups respectively reported having this information compared to 42% of women in the no contact group. Overall, the level of information of the direct contact group was better than that of the no contact group.

**Table 3:** Knowledge/information about general health issues (n = 1,140)

**Variables**	**Direct contact with the ICC, % (n = 380)**	**In-direct contact with the ICC, % (n = 380)**	**No contact with the ICC, % (n = 380)**	**Chi square**^**a **^**p-value**
**Information about:**				<0.001
Dangers of self-medication				
Yes	76.6	77.1	66.6	
No	23.4	22.9	33.4	
Use of disposal needle for injection				<0.001
Yes	83.4	51.8	52.9	
No	16.6	48.2	47.1	
Causes of hepatitis				0.12
Yes	53.7	51.8	58.9	
No	46.3	48.2	41.1	
Dangers of treatment with quacks				<0.001
Yes	52.9	31.8	43.9	
No	47.1	68.2	56.1	
Importance of washing hands after dispensing pesticide				<0.001
Yes	91.8	83.9	85.0	
No	8.2	16.1	15.0	
Importance of washing hands before eating				0.16
Yes	97.9	95.8	97.7	
No	2.1	4.2	2.3	
Importance of boiling drinking water				<0.001
Yes	88.9	73.9	68.9	
No	11.1	26.1	31.1	
Harmful effects of spousal violence				<0.001
Yes	48.9	32.1	27.8	
No	51.1	67.9	72.2	
Importance of garbage disposal				<0.01
Yes	52.9	48.9	41.8	
No	47.1	51.1	58.2	
Cleaning teeth daily				<0.05
Yes	62.9	55.8	53.2	
No	37.1	44.2	46.8	

^a^Fisher exact test.

## Discussion

As in other developing countries, in Pakistan access to information is not evenly distributed, and neither is the capacity to understand and utilize the available information. For instance, some sections of society have disproportionally high access to knowledge and information and also use that information to their socioeconomic advantage [34,35]. Conversely, illiterates and the poor, including women, old, and marginalized people have lesser access to information, and as a result they become further isolated and excluded [10,19]. Recent research has shown that the digitally driven flow of information, instead of bridging the information gap, is further widening it [10,36].

In Pakistan, the importance of information for human development and poverty alleviation is not yet fully recognized. Despite the fact that information is essential for the maintenance of health and well-being, the official Department of Health has made no systematic arrangements to improve the level of health information. Women, irrespective of location, need information on family health, food and nutrition, family planning, and child education as well as opportunities to become involved in socio-economic growth [23], but a majority of rural Pakistani women are deprived of access to knowledge and information [10,23,37].

The information deficit tends to cause countless and lifelong deprivations such as poor health, the inability to earn independently or protect their basic health rights, and a lack of reproductive autonomy. In essence, women who are deprived of knowledge and information cannot help themselves and remain trapped in a vicious cycle of poverty [38]. This could be one reason why Pakistan is ranked 134th out of 135 countries in terms of the gender gap. This means that Pakistani women are considerably below the global average on four sub-indexes: economic participation, educational attainment, health and survival, and political empowerment [39].

For the provision of this information, there is a need for the democratization of knowledge and an inclusive approach to enhance access to information. But information systems do not operate in a vacuum; the provision of information entails some sort of social change and may upset the existing political order and gender relations. With our experience of the ICC, we noted that provision of information to women was a sensitive and challenging endeavor, particularly in rural areas. The local culture was very cautious and skeptical about any possible change in the role and status of women. In a strict patriarchal regime such as Pakistan, women are only allowed to access “culturally correct and dramatically useful” information.

The experience of the ICC showed that health-related information was abundant and easily available. But the real problem was the absence of a cultural mechanism that made sense of this information for common people, especially for rural women. For this, a culturally relevant information processing system needs to be developed. A system that connects people to each other despite barriers of time, distance, and literacy is in high demand among poor rural communities [38]. Such a system can only be developed with the active participation and engagement of the end-users, the village women.

Despite various limitations, the greatest success of the ICC was to provide an opportunity for ordinary poor women to become engaged in the process of discourse and dialogue about health-related information on their doorstep. The encouraging aspect is that most of the local women, illiterates included, were fully aware of the importance of information and became proactively involved in the activities of the ICC.

The results of the evaluation of the functioning of the ICC showed that the women who had direct or indirect contact with it had better levels of information than the no contact group. This indicates some success in improving the quality and quantity of health-related information for women. Nonetheless, it does not necessarily mean that the no contact group lacked information just because of their lack of connection with the ICC. There is a possibility that these women might belong to families that place harsh restrictions on their mobility or establishing outside contact with other women.

### Strengths of the ICC

The functioning of the ICC brought many unexpected outcomes. Firstly, it exposed the redundancy and irrelevance of information available on the “official websites of government departments”. The participants of the ICC termed standard “one-size-fits-all” information useless for them. The ICC provided the government functionaries a platform where they interacted with “common women” in an informal environment and they knew the worth of their “information products”. In this way, ICC helped the government functionaries to improve their information provision services.

Secondly, the ICC tried to bridge the conceptual and cognitive gap between modern medical system and indigenous medical system. It may be noted that the government health department provides health-related scientific information in the areas of reproductive health, sanitation, and prevention of epidemics. But in villages, where the literacy level is quite low, women usually believe in the indigenous medical system, which is different from the modern bio-medical system. Since both systems are grounded on different language, terminology and world-view, the transfer of knowledge/information from one system to another needs a trusted and credible local institution. The ICC successfully performed this function.

Thirdly, the health-related information was just printed or presented and nobody was there to address the questions, concerns, and fears of women about its applicability and relevance to their health needs. It was just one-way flow of information. For instance, in the local culture, there was a tradition to give a bath to an infant immediately after birth. But doctors viewed this as a dangerous practice because the infant is exposed to a sudden change of temperature. Now, just giving the information that “one should not give a bath to infants immediate after delivery” is not enough, unless women are thoroughly educated about the harmful effects of this practice in a culturally understandable way. So, it is not just a question of giving information, there needs to be a comprehensive strategy to change the health-related behavior of women. At ICC, the participants comprehensively and frankly discussed information. Such live and locally understandable debates at the ICC thoroughly addressed the women’s concerns and removed their deep-seated fears about what might happen if they opted to abandon their traditional health beliefs and practices.

### Limitations of the functioning of ICC

Despite various strengths of the ICC, there were some structural and procedural limitations of the functioning of ICC. Firstly, there was a serious shortage of health-related information which could be relevant and applicable for the local population. In some situations, women were disappointed when the ICC management was unable to provide them appropriate information. It may be noted that ICC had no mandate to generate health-related technical information which otherwise was not available in lucid local language (e.g., information related to prevention and treatment of reproductive tract infections). Given the paucity of relevant and updated information, sometimes, the ICC management had to refer these women to distant government institutions and the women were reluctant to contact these organizations because of various cultural and infrastructural inhibitions. It was one of the major limitations of the ICC.

Secondly, sometimes, cultural norms of gender segregation undermined the functioning of ICC. For example, many local experts and functionaries of government departments were male. When they visited the ICC to disseminate information, some conservative local leaders raised objections to these “co-gatherings” and considered them contrary to the cultural norms and religious tradition.

Thirdly, frequent breakdown of electricity, disruption in internet connectivity, high temperature in summer season and general feeling of insecurity undermined the smooth functioning of the ICC. For instance, in the absence of electricity (in summer, daily breakdown of electricity was 8–12 hours) it was simply not possible to continue information providing sessions at ICC because of hot and humid weather and stoppage of ICT tools and devices.

### Strengths and limitations of the study

The major strength of this study is that the ICC operations were embedded in the real life of the women within their institutional and community contexts. The women’s perspectives on functioning of the ICC were drawn by direct, constant, and engaging interactions in the natural setting. Hence, this pilot study has good prospects of replication in similar settings to improve health information of rural women.

Nonetheless, short duration of the project was its limitation. Additional positive impacts can be assumed by enriching the community health information system for a longer duration. Similarly, the evaluation phase of this study has some limitations. Women in the direct and no contact group greatly differ in respect to their socio-demographic characteristics, so it was difficult to discern accurate results regarding their knowledge about reproductive and general health issues. Additionally, the survey was cross-sectional in nature: hence, we were unable to check that whether the health information imparted through the ICC was followed.

## Conclusions

In short, the experience of the ICC deepened our understanding of the health-related information needs of rural women in Pakistan. Based on the experiences gained from the ICC, we suggest that the provision of standard one-way information is not enough. In order to make women capable of utilizing health-related information, Pakistani policy makers, academia, and development experts need to make concerted efforts to create information according to local needs and demands. It is not safe to assume that the technology will automatically empower women and improve their access to health-related information. Technology does help, but society needs to develop a social mechanism whereby women become engaged and involved in information discourse and dialogue at grass-roots level.

There is a paucity of research in Pakistan on the development of health-related information systems which could be useful for local population. For this, the first step is to establish community-based and community-owned information dissemination institutions. The ICC was a step in this direction. It is also important that Pakistani policy makers understand the complexity and multidimensionality of the information needs of various sections of the society and increasing plurality of information provision sources. It is suggested to compare the effectiveness of information received through different sources of information (e.g., ICTs, print media, radio and TV) and to determine the relative impact of different sources of information on behavior-change of people. Future research ought to focus on the main health concerns of rural women, their health information needs, and the barriers to getting health-related information within their cultural and structural limitations.

## Competing interests

The authors declare that they have no competing interests.

## Authors’ contributions

MZZ conceptualized the study. RZ and MZZ carried out the study and participated in the field research. They also performed the qualitative and statistical analysis. SQ and FF contributed to the interpretation of data. RZ, MZZ, SQ and FF drafted the manuscript and contributed in revising the manuscript. All authors read and approved the final manuscript.

## Pre-publication history

The pre-publication history for this paper can be accessed here:

http://www.biomedcentral.com/1472-6874/14/105/prepub
